# 4-{4-[(4-Oxoquinazolin-3-yl)meth­yl]-1*H*-1,2,3-triazol-1-yl}butyl acetate

**DOI:** 10.1107/S1600536811045600

**Published:** 2011-11-05

**Authors:** Abdelaaziz Ouahrouch, Moha Taourirte, Hassan B. Lazrek, Mohamed El Azhari, Mohamed Saadi, Lahcen El Ammari

**Affiliations:** a Laboratoire de Chimie Bio-organique et Macromoléculaire, Faculté des Sciences et Techniques Guéliz, Marrakech, Morocco; b Unité de Chimie Biomoléculaire et Médicinale, Faculté des Sciences Semlalia, Marrakech, Morocco; c Laboratoire de la Matière Condensée et des Nanostructures, Faculté des Sciences et Techniques Guéliz, Marrakech, Morocco; d Laboratoire de Chimie du Solide Appliquée, Faculté des Sciences, Université Mohammed V-Agdal, Avenue Ibn Battouta, BP 1014, Rabat, Morocco

## Abstract

In the heterocyclic title compound, C_17_H_19_N_5_O_3_, the quinazolinone ring system forms a dihedral angle of 67.22 (7)° with the triazole ring. The butyl acetate group has a non-linear conformation, with an alternation of synclinal and anti­periplanar torsion angles [N—C—C—C = 58.5 (2)°, C—C—C—C = 170.72 (19)° and C—C—C—O = −65.9 (3)°]. The crystal structure features inter­molecular C—H⋯N and C—H⋯O non-classical hydrogen bonds, building an infinite one-dimensional network along the [100] direction.

## Related literature

For details of the synthesis, see: Krim *et al.* (2009 ▶); Mani Chandrika *et al.* (2010 ▶). For background to the biological activity of quinazolinone derivatives, see: Alvarez *et al.* (1994 ▶); Xu *et al.* (2007 ▶); Apfel *et al.* (2001 ▶); Tobe *et al.* (2003 ▶); Fung-Tome *et al.* (1998 ▶); Genin *et al.* (2000 ▶).
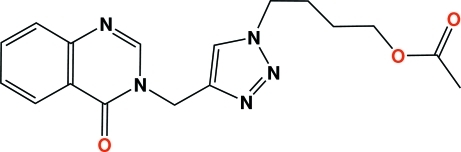


## Experimental

### 

#### Crystal data

C_17_H_19_N_5_O_3_
*M* *_r_* = 341.37
Orthorhombic, 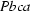

*a* = 10.2546 (4) Å
*b* = 8.7643 (3) Å
*c* = 37.5434 (13) Å
*V* = 3374.2 (2) Å^3^
*Z* = 8
Mo *K*α radiation
μ = 0.10 mm^−1^
*T* = 296 K
0.46 × 0.35 × 0.18 mm

#### Data collection

Bruker X8 APEXII diffractometer
19997 measured reflections
3676 independent reflections
2830 reflections with *I* > 2σ(*I*)
*R*_int_ = 0.031

#### Refinement

*R*[*F* ^2^ > 2σ(*F* ^2^)] = 0.047
*wR*(*F* ^2^) = 0.133
*S* = 1.05
3676 reflections
226 parameters
H-atom parameters constrained
Δρ_max_ = 0.31 e Å^−3^
Δρ_min_ = −0.17 e Å^−3^

### 

Data collection: *APEX2* (Bruker, 2005 ▶); cell refinement: *SAINT* (Bruker, 2005 ▶); data reduction: *SAINT*; program(s) used to solve structure: *SHELXS97* (Sheldrick, 2008 ▶); program(s) used to refine structure: *SHELXL97* (Sheldrick, 2008 ▶); molecular graphics: *ORTEP-3 for Windows* (Farrugia,1997 ▶); software used to prepare material for publication: *WinGX* (Farrugia, 1999 ▶).

## Supplementary Material

Crystal structure: contains datablock(s) I, global. DOI: 10.1107/S1600536811045600/kj2192sup1.cif

Structure factors: contains datablock(s) I. DOI: 10.1107/S1600536811045600/kj2192Isup2.hkl

Supplementary material file. DOI: 10.1107/S1600536811045600/kj2192Isup3.cml

Additional supplementary materials:  crystallographic information; 3D view; checkCIF report

## Figures and Tables

**Table 1:** Hydrogen-bond geometry (Å, °)

*D*—H⋯*A*	*D*—H	H⋯*A*	*D*⋯*A*	*D*—H⋯*A*
C8—H8⋯N4^i^	0.93	2.58	3.180 (2)	123
C9—H9*A*⋯N4^i^	0.97	2.58	3.418 (2)	145
C11—H11⋯N3^i^	0.93	2.57	3.359 (2)	143
C12—H12*B*⋯O1^i^	0.97	2.51	3.433 (2)	160

Symmetry code: (i) 
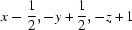
.

**Fig. 1.**
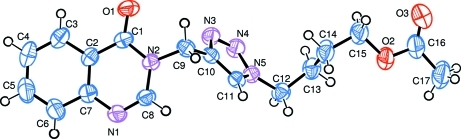
: Plot of the title compound with the atom-labelling scheme. Displacement ellipsoids are drawn at the 50% probability level. H atoms are represented as small circles.

**Fig. 2.**
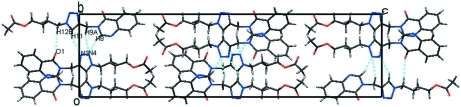
: Partiel plot of the title compound, showing two molecules linked through C—H···N and C–H···O non classical hydrogen bonds (dashed lines).

## supplementary crystallographic information

### Comment

The most interesting classes of compounds possess a wide spectrum of biological activity, we can found the quinazolinones derivatives (Apfel *et al.* (2001)). They are broadly used in pharmaceuticals and agrochemicals (Tobe *et al.* (2003)). For example, diseases of agriculture are treated by fungicide fluquinconazole (Xu *et al.* (2007)). Also, the most encountered heterocycle in medicinal chemistry, five-membered nitrogen heterocycles, they have an important part in biological systems. Among these, 1,2,3-triazole heterocycles have several biological activities, including anti-HIV (Alvarez *et al.* (1994)), anti-fungal, and antimicrobial activities (Fung-Tome *et al.* (1998); Genin *et al.* (2000)). 1,2,3-triazoles are useful products in chemistry, stable metabolism, stable to moisture, oxygen, light, and also metabolism in the body.

The molecule of the title compound is built up from two fused six-membered rings linked to a five-membered ring which is connected to butyl acetate group as shown in Fig.1. The quinazolinone ring is almost planar, with a maximum deviation of 0.0502 (14) Å for O1. The dihedral angle between the quinazolinone mean plane and the triazol ring amount to 67.22 (7)°. The butylacetate group has a non-linear conformation, with an alternation of synclinal and antiperiplanar torsion angles N5—C12—C13—C14 = 58.52 (23)°, C12—C13—C14—C15 = 170.72 (19)° and C13 - C14 - C15 - O2 = -65.91 (28)°.

An intermolecular C–H···N and C–H···O non classic hydrogen bonds, building an infinite one-dimensional network along [1 0 0] direction ensure the cohesion of the crystal structure as schown in Fig.2 and Table 1.

### Experimental

The title compound was prepared by cycloaddition of propargylated quinazolinone and azide under microwave conditions with CuI as catalyst and without solvent. The product was obtained with quantitative yield (96%) and short reaction time (Mani Chandrika *et al.* (2010); Krim *et al.* (2009)). The crude product was purified passing through a column packed with silica gel. Crystal suitable for X-ray analysis was obtained by slow evaporation of a methanol / methylene chloride (5:95 *v*/*v*) solution. The melting point of this crystal is in the range of 353–354 K.

### Refinement

The structure is solved by direct method technique and refined by full-matrix least-squares using *SHELXS97* and *SHELXL97* program packages. H atoms were located in a difference map and treated as riding with C—H = 0.97 Å and 0.93 Å for –CH_2_– and aromatic CH respectively. All H atoms with *U*_iso_(H) = 1.2 *U*_eq_ (aromatic, methylene) and *U*_iso_(H) = 1.5 *U*_eq_ for the methyl group.

### Crystal data

**Table:**

C_17_H_19_N_5_O_3_	*F*(000) = 1440
*M**_r_* = 341.37	*D*_x_ = 1.344 Mg m^−^^3^
Orthorhombic, *P**b**c**a*	Mo *K*α radiation, λ = 0.71073 Å
Hall symbol: -p 2ac 2ab	Cell parameters from 3676 reflections
*a* = 10.2546 (4) Å	θ = 2.3–27.0°
*b* = 8.7643 (3) Å	µ = 0.10 mm^−^^1^
*c* = 37.5434 (13) Å	*T* = 296 K
*V* = 3374.2 (2) Å^3^	Parallelepiped, colourless
*Z* = 8	0.46 × 0.35 × 0.18 mm

### Data collection

**Table:**

Bruker X8 APEXII diffractometer	2830 reflections with *I* > 2σ(*I*)
Radiation source: fine-focus sealed tube	*R*_int_ = 0.031
graphite	θ_max_ = 27.0°, θ_min_ = 2.3°
φ and ω scans	*h* = −12→13
19997 measured reflections	*k* = −10→11
3676 independent reflections	*l* = −47→47

### Refinement

**Table:**

Refinement on *F*^2^	Primary atom site location: structure-invariant direct methods
Least-squares matrix: full	Secondary atom site location: difference Fourier map
*R*[*F*^2^ > 2σ(*F*^2^)] = 0.047	Hydrogen site location: difference Fourier map
*wR*(*F*^2^) = 0.133	H-atom parameters constrained
*S* = 1.05	*w* = 1/[σ^2^(*F*_o_^2^) + (0.0594*P*)^2^ + 1.1103*P*] where *P* = (*F*_o_^2^ + 2*F*_c_^2^)/3
3676 reflections	(Δ/σ)_max_ = 0.001
226 parameters	Δρ_max_ = 0.31 e Å^−^^3^
0 restraints	Δρ_min_ = −0.17 e Å^−^^3^

### Special details

**Table:**

Geometry. All s.u.'s (except the s.u. in the dihedral angle between two l.s. planes) are estimated using the full covariance matrix. The cell s.u.'s are taken into account individually in the estimation of s.u.'s in distances, angles and torsion angles; correlations between s.u.'s in cell parameters are only used when they are defined by crystal symmetry. An approximate (isotropic) treatment of cell s.u.'s is used for estimating s.u.'s involving l.s. planes.
Refinement. Refinement of *F*^2^ against all reflections. The weighted *R*-factor *wR* and goodness of fit *S* are based on *F*^2^, conventional *R*-factors *R* are based on *F*, with *F* set to zero for negative *F*^2^. The threshold expression of *F*^2^ > 2σ(*F*^2^) is used only for calculating *R*-factors(gt) *etc*. and is not relevant to the choice of reflections for refinement. *R*-factors based on *F*^2^ are statistically about twice as large as those based on *F*, and *R*- factors based on all data will be even larger.

### Fractional atomic coordinates and isotropic or equivalent isotropic displacement parameters (Å^2^)

**Table:**

	*x*	*y*	*z*	*U*_iso_*/*U*_eq_	
C1	0.44375 (15)	0.12932 (18)	0.58669 (4)	0.0395 (4)	
C2	0.42450 (16)	0.18373 (19)	0.62292 (4)	0.0415 (4)	
C3	0.5106 (2)	0.1412 (2)	0.65006 (5)	0.0570 (5)	
H3	0.5802	0.0765	0.6451	0.068*	
C4	0.4924 (3)	0.1948 (3)	0.68401 (6)	0.0746 (7)	
H4	0.5492	0.1655	0.7021	0.090*	
C5	0.3899 (3)	0.2922 (3)	0.69155 (6)	0.0766 (7)	
H5	0.3786	0.3281	0.7146	0.092*	
C6	0.3051 (2)	0.3361 (3)	0.66543 (5)	0.0640 (5)	
H6	0.2369	0.4022	0.6708	0.077*	
C7	0.32078 (17)	0.2818 (2)	0.63062 (4)	0.0452 (4)	
C8	0.25014 (17)	0.27360 (19)	0.57352 (4)	0.0447 (4)	
H8	0.1905	0.3024	0.5561	0.054*	
C9	0.34796 (17)	0.11979 (18)	0.52617 (4)	0.0420 (4)	
H9A	0.2615	0.0820	0.5205	0.050*	
H9B	0.4081	0.0347	0.5244	0.050*	
C10	0.38543 (15)	0.23769 (18)	0.49936 (4)	0.0370 (3)	
C11	0.30920 (15)	0.32006 (17)	0.47674 (4)	0.0379 (4)	
H11	0.2189	0.3170	0.4747	0.045*	
C12	0.36418 (18)	0.5147 (2)	0.42924 (4)	0.0481 (4)	
H12A	0.3987	0.6141	0.4356	0.058*	
H12B	0.2705	0.5246	0.4266	0.058*	
C13	0.4222 (2)	0.4655 (2)	0.39423 (5)	0.0545 (5)	
H13A	0.4008	0.5414	0.3764	0.065*	
H13B	0.5164	0.4636	0.3966	0.065*	
C14	0.3778 (3)	0.3121 (2)	0.38106 (5)	0.0669 (6)	
H14A	0.4119	0.2336	0.3967	0.080*	
H14B	0.2833	0.3075	0.3821	0.080*	
C15	0.4210 (3)	0.2797 (3)	0.34367 (6)	0.0885 (9)	
H15A	0.5148	0.2914	0.3418	0.106*	
H15B	0.3986	0.1758	0.3372	0.106*	
C16	0.3964 (3)	0.3897 (3)	0.28651 (6)	0.0746 (7)	
C17	0.3213 (3)	0.4995 (3)	0.26464 (6)	0.0899 (8)	
H17A	0.3770	0.5419	0.2466	0.135*	
H17B	0.2889	0.5799	0.2796	0.135*	
H17C	0.2494	0.4478	0.2536	0.135*	
N1	0.23250 (15)	0.32838 (18)	0.60483 (4)	0.0506 (4)	
N2	0.34803 (13)	0.17690 (15)	0.56310 (3)	0.0371 (3)	
N3	0.51154 (13)	0.27696 (17)	0.49359 (4)	0.0455 (3)	
N4	0.51522 (13)	0.38114 (18)	0.46833 (4)	0.0477 (4)	
N5	0.39226 (12)	0.40645 (15)	0.45805 (3)	0.0390 (3)	
O1	0.53465 (12)	0.04938 (16)	0.57675 (4)	0.0590 (4)	
O2	0.35600 (17)	0.38607 (17)	0.32010 (3)	0.0707 (4)	
O3	0.4835 (3)	0.3124 (3)	0.27576 (5)	0.1304 (9)	

### Atomic displacement parameters (Å^2^)

**Table:**

	*U*^11^	*U*^22^	*U*^33^	*U*^12^	*U*^13^	*U*^23^
C1	0.0329 (8)	0.0366 (8)	0.0489 (9)	−0.0002 (7)	0.0029 (7)	0.0085 (7)
C2	0.0403 (9)	0.0405 (8)	0.0438 (8)	−0.0057 (7)	−0.0016 (7)	0.0099 (7)
C3	0.0551 (12)	0.0599 (11)	0.0561 (11)	0.0029 (9)	−0.0081 (9)	0.0153 (9)
C4	0.0828 (16)	0.0923 (17)	0.0488 (11)	−0.0010 (14)	−0.0203 (11)	0.0142 (11)
C5	0.0904 (18)	0.0976 (18)	0.0420 (10)	0.0011 (15)	−0.0043 (11)	−0.0044 (11)
C6	0.0669 (13)	0.0765 (14)	0.0487 (10)	0.0041 (11)	0.0026 (9)	−0.0096 (10)
C7	0.0430 (10)	0.0475 (9)	0.0451 (9)	−0.0034 (8)	0.0010 (7)	−0.0002 (7)
C8	0.0382 (8)	0.0458 (9)	0.0500 (9)	0.0065 (7)	−0.0061 (8)	−0.0026 (7)
C9	0.0435 (9)	0.0384 (8)	0.0441 (8)	−0.0019 (7)	0.0025 (7)	−0.0045 (7)
C10	0.0340 (8)	0.0409 (8)	0.0361 (7)	−0.0004 (7)	0.0020 (6)	−0.0072 (6)
C11	0.0300 (8)	0.0425 (8)	0.0411 (8)	−0.0030 (7)	0.0007 (6)	−0.0070 (7)
C12	0.0523 (10)	0.0430 (9)	0.0491 (9)	0.0009 (8)	−0.0028 (8)	0.0038 (7)
C13	0.0651 (12)	0.0525 (11)	0.0460 (10)	−0.0046 (9)	0.0008 (9)	0.0100 (8)
C14	0.1040 (18)	0.0491 (11)	0.0476 (10)	0.0043 (11)	−0.0131 (11)	0.0057 (8)
C15	0.150 (3)	0.0665 (14)	0.0493 (11)	0.0397 (16)	−0.0138 (14)	0.0002 (10)
C16	0.0973 (19)	0.0825 (15)	0.0439 (11)	0.0087 (14)	−0.0073 (11)	−0.0063 (10)
C17	0.113 (2)	0.1042 (19)	0.0524 (12)	0.0041 (17)	−0.0111 (13)	0.0192 (12)
N1	0.0435 (8)	0.0576 (9)	0.0508 (8)	0.0111 (7)	−0.0049 (7)	−0.0090 (7)
N2	0.0348 (7)	0.0363 (6)	0.0401 (7)	0.0000 (5)	0.0010 (5)	0.0017 (5)
N3	0.0336 (7)	0.0573 (9)	0.0457 (8)	0.0015 (6)	0.0012 (6)	0.0031 (7)
N4	0.0343 (8)	0.0620 (9)	0.0470 (8)	−0.0009 (7)	0.0032 (6)	0.0050 (7)
N5	0.0343 (7)	0.0451 (7)	0.0377 (7)	−0.0003 (6)	−0.0003 (5)	−0.0024 (6)
O1	0.0455 (7)	0.0673 (8)	0.0642 (8)	0.0195 (7)	0.0041 (6)	0.0048 (7)
O2	0.0997 (12)	0.0707 (9)	0.0417 (7)	0.0180 (9)	−0.0043 (7)	0.0062 (6)
O3	0.166 (2)	0.170 (2)	0.0556 (10)	0.0787 (19)	0.0028 (12)	−0.0119 (12)

### Geometric parameters (Å, °)

**Table:**

C1—O1	1.224 (2)	C11—N5	1.338 (2)
C1—N2	1.386 (2)	C11—H11	0.9300
C1—C2	1.455 (2)	C12—N5	1.467 (2)
C2—C7	1.398 (2)	C12—C13	1.506 (2)
C2—C3	1.399 (2)	C12—H12A	0.9700
C3—C4	1.371 (3)	C12—H12B	0.9700
C3—H3	0.9300	C13—C14	1.503 (3)
C4—C5	1.383 (3)	C13—H13A	0.9700
C4—H4	0.9300	C13—H13B	0.9700
C5—C6	1.366 (3)	C14—C15	1.499 (3)
C5—H5	0.9300	C14—H14A	0.9700
C6—C7	1.400 (2)	C14—H14B	0.9700
C6—H6	0.9300	C15—O2	1.448 (2)
C7—N1	1.387 (2)	C15—H15A	0.9700
C8—N1	1.283 (2)	C15—H15B	0.9700
C8—N2	1.371 (2)	C16—O3	1.191 (3)
C8—H8	0.9300	C16—O2	1.328 (3)
C9—N2	1.474 (2)	C16—C17	1.481 (3)
C9—C10	1.493 (2)	C17—H17A	0.9600
C9—H9A	0.9700	C17—H17B	0.9600
C9—H9B	0.9700	C17—H17C	0.9600
C10—N3	1.356 (2)	N3—N4	1.317 (2)
C10—C11	1.361 (2)	N4—N5	1.3372 (19)
			
O1—C1—N2	121.10 (16)	C13—C12—H12B	109.1
O1—C1—C2	125.16 (15)	H12A—C12—H12B	107.9
N2—C1—C2	113.74 (14)	C14—C13—C12	115.05 (17)
C7—C2—C3	119.57 (17)	C14—C13—H13A	108.5
C7—C2—C1	119.89 (15)	C12—C13—H13A	108.5
C3—C2—C1	120.53 (16)	C14—C13—H13B	108.5
C4—C3—C2	120.0 (2)	C12—C13—H13B	108.5
C4—C3—H3	120.0	H13A—C13—H13B	107.5
C2—C3—H3	120.0	C15—C14—C13	112.8 (2)
C3—C4—C5	120.36 (19)	C15—C14—H14A	109.0
C3—C4—H4	119.8	C13—C14—H14A	109.0
C5—C4—H4	119.8	C15—C14—H14B	109.0
C6—C5—C4	120.7 (2)	C13—C14—H14B	109.0
C6—C5—H5	119.6	H14A—C14—H14B	107.8
C4—C5—H5	119.6	O2—C15—C14	108.32 (18)
C5—C6—C7	120.1 (2)	O2—C15—H15A	110.0
C5—C6—H6	120.0	C14—C15—H15A	110.0
C7—C6—H6	120.0	O2—C15—H15B	110.0
N1—C7—C2	122.22 (15)	C14—C15—H15B	110.0
N1—C7—C6	118.47 (17)	H15A—C15—H15B	108.4
C2—C7—C6	119.30 (17)	O3—C16—O2	122.8 (2)
N1—C8—N2	126.59 (15)	O3—C16—C17	124.8 (2)
N1—C8—H8	116.7	O2—C16—C17	112.3 (2)
N2—C8—H8	116.7	C16—C17—H17A	109.5
N2—C9—C10	113.52 (13)	C16—C17—H17B	109.5
N2—C9—H9A	108.9	H17A—C17—H17B	109.5
C10—C9—H9A	108.9	C16—C17—H17C	109.5
N2—C9—H9B	108.9	H17A—C17—H17C	109.5
C10—C9—H9B	108.9	H17B—C17—H17C	109.5
H9A—C9—H9B	107.7	C8—N1—C7	115.95 (15)
N3—C10—C11	108.27 (14)	C8—N2—C1	121.49 (13)
N3—C10—C9	121.94 (14)	C8—N2—C9	118.55 (13)
C11—C10—C9	129.78 (15)	C1—N2—C9	119.94 (13)
N5—C11—C10	105.17 (13)	N4—N3—C10	108.56 (13)
N5—C11—H11	127.4	N3—N4—N5	107.21 (13)
C10—C11—H11	127.4	N4—N5—C11	110.78 (13)
N5—C12—C13	112.37 (14)	N4—N5—C12	120.34 (13)
N5—C12—H12A	109.1	C11—N5—C12	128.86 (14)
C13—C12—H12A	109.1	C16—O2—C15	116.87 (19)
N5—C12—H12B	109.1		
			
O1—C1—C2—C7	176.30 (16)	C2—C7—N1—C8	1.3 (3)
N2—C1—C2—C7	−3.4 (2)	C6—C7—N1—C8	−179.03 (18)
O1—C1—C2—C3	−2.4 (3)	N1—C8—N2—C1	−1.4 (3)
N2—C1—C2—C3	177.87 (15)	N1—C8—N2—C9	176.86 (17)
C7—C2—C3—C4	0.5 (3)	O1—C1—N2—C8	−176.14 (16)
C1—C2—C3—C4	179.16 (19)	C2—C1—N2—C8	3.6 (2)
C2—C3—C4—C5	−0.6 (3)	O1—C1—N2—C9	5.6 (2)
C3—C4—C5—C6	0.2 (4)	C2—C1—N2—C9	−174.61 (13)
C4—C5—C6—C7	0.4 (4)	C10—C9—N2—C8	72.82 (19)
C3—C2—C7—N1	179.81 (17)	C10—C9—N2—C1	−108.91 (16)
C1—C2—C7—N1	1.1 (3)	C11—C10—N3—N4	0.31 (18)
C3—C2—C7—C6	0.2 (3)	C9—C10—N3—N4	179.09 (14)
C1—C2—C7—C6	−178.54 (17)	C10—N3—N4—N5	−0.49 (18)
C5—C6—C7—N1	179.7 (2)	N3—N4—N5—C11	0.50 (18)
C5—C6—C7—C2	−0.6 (3)	N3—N4—N5—C12	−178.25 (14)
N2—C9—C10—N3	79.07 (19)	C10—C11—N5—N4	−0.30 (17)
N2—C9—C10—C11	−102.43 (19)	C10—C11—N5—C12	178.31 (15)
N3—C10—C11—N5	0.00 (17)	C13—C12—N5—N4	63.2 (2)
C9—C10—C11—N5	−178.66 (15)	C13—C12—N5—C11	−115.34 (19)
N5—C12—C13—C14	58.5 (2)	O3—C16—O2—C15	−1.4 (4)
C12—C13—C14—C15	170.72 (19)	C17—C16—O2—C15	178.6 (2)
C13—C14—C15—O2	−65.9 (3)	C14—C15—O2—C16	170.4 (2)
N2—C8—N1—C7	−1.3 (3)		

### Hydrogen-bond geometry (Å, °)

**Table:**

*D*—H···*A*	*D*—H	H···*A*	*D*···*A*	*D*—H···*A*
C8—H8···N4^i^	0.93	2.58	3.180 (2)	123.
C9—H9A···N4^i^	0.97	2.58	3.418 (2)	145.
C11—H11···N3^i^	0.93	2.57	3.359 (2)	143.
C12—H12B···O1^i^	0.97	2.51	3.433 (2)	160.

Symmetry codes: (i) *x*−1/2, −*y*+1/2, −*z*+1.
